# A Textbook on Diseases of Infancy and Childhood

**Published:** 1902-12

**Authors:** 


A Textbook on Diseases of Infancy and Childhood. For the use of students and practitioners of medicine. By Henry Koplik, M. D., Attending Pediatrist, Mt. Sinai Hospital, New York. Octavo, pp. 675. Illustrated with 169 engravings and 30 plates in color and monochrome. Philadelphia and New York: Lea Brothers & Co., 1902. [Price, cloth, $5.00; leather, $6.00 net.]

In taking up this book to pass judgment upon it one must have in mind its scope and limitations. It does not aim to cover the whole field of pediatrics, as does Holt's Diseases of Infancy and Childhood, and it cannot therefore justly be compared with such a work. It does not deal at all, or only incidentally, with the surgical diseases of childhood. Diseases which do not differ materially in their behavior in childhood from that in adult life are not considered at length and rare conditions are but briefly described.

The subjects which fall within the intent of the work are in the main satisfactorily handled. Morbid anatomy is not dwelt upon very fully and in this feature one feels that the book is somewhat lacking. The chapter on infant feeding might have been made longer with profit to the reader. To point out now some of the commendable features one may say, not to mention others perhaps equally good, that the chapters on drugs and other therapeutic means, diseases of the heart, contagious diseases, the blood, and scrofulosis, are excellent. In the matter of treatment the book is altogether commendable. One feels that the author is an observing, original and successful therapist.

The illustrations are numerous and good. Many of them are new. There are a few colored plates, among them an excellent one showing Koplik's spots, the pathognomonic symptom of measles first described by Dr. Koplik.

Dr. Koplik has reinforced his own practical knowledge of his subject by drawing largely from both American and foreign literature, as the bibliographies appended to each chapter testify. He announces in the preface that his purpose has been to afford to students and practitioners a practical guide and textbook and it must be conceded that he has accomplished this.

It is of local interest to note that the work is dedicated to the author's preceptors, Drs. Francis Delafield and Matthew D. Mann. M. J. F.

